# Comparison of Perinatal and Maternal Outcomes in Borderline Versus Normal Amniotic Fluid Index in a Tertiary Care Center in Odisha: An Observational Prospective Study

**DOI:** 10.7759/cureus.19876

**Published:** 2021-11-24

**Authors:** Anuradha Vyas, G Prasanna, Sudarshan Dash, Sudhansu Rath

**Affiliations:** 1 Obstetrics and Gynaecology, Kalinga Institute of Medical Sciences, Kalinga Institute of Industrial Technology, Bhubaneswar, IND

**Keywords:** nicu, fetal distress, perinatal outcomes, borderline afi, amniotic fluid

## Abstract

Background

Amniotic fluid is a protective fluid in the amniotic sac of a gravid uterus that serves many crucial functions by becoming part of an indicator of a functioning fetoplacental unit during the intrauterine life of a fetus. The most commonly used method for measuring amniotic fluid is the amniotic fluid index (AFI). In this study, we aimed to investigate the perinatal and maternal outcomes in borderline AFI versus normal AFI.

Methodology

This observational prospective study included 200 pregnant women who were admitted to Pradyumna Bal Memorial Hospital, Bhubaneswar from September 2019 to February 2021. Women with singleton pregnancy in their third trimester were enrolled in this study after applying inclusion and exclusion criteria. Of the included women, 100 were cases with borderline AFI, and 100 were control with normal AFI. Fetal and maternal outcomes were compared between the two groups. Data analysis was done using SPSS version 23 (IBM Corp., Armonk, NY, USA).

Results

Maternal outcomes such as preterm delivery, meconium-stained liquor, and lower segment cesarean section in women with borderline AFI were significantly higher (p ≤ 0.001). The borderline AFI group had a higher rate of perinatal complications such as Apgar score of <7 (p = 0.001), respiratory distress syndrome (p = 0.001), neonatal intensive care unit admission (p <0.001), intrauterine growth restriction (p < 0.001), and low birth weight (p < 0.001).

Conclusions

The borderline AFI group was associated with adverse perinatal and maternal outcomes which were significantly higher in this group compared to the control group. Therefore, patients with borderline AFI should be monitored carefully during the antepartum and intrapartum period.

## Introduction

Amniotic fluid is the protective fluid contained in the amniotic sac of a gravid uterus. This fluid acts as a cushion for the growing fetus and facilitates the exchange of nutrients, water, and biochemical products between the mother and the fetus. Amniotic fluid is the ultrafiltrate of maternal plasma and passes through the fetal membrane through osmotic and hydrostatic forces. When fetal kidneys become functional at approximately 16 weeks, fetal urine also contributes to the fluid. Although amniotic fluid is mainly removed by fetal swallowing, some amount of fluid is absorbed by the fetal skin as well.

Amniotic fluid volume is related to gestational age. In this study, we excluded preterm premature rupture of membrane (PPROM) and premature rupture of membrane (PROM) cases and only included pregnancies with intact fetal membranes. Amniotic fluid measures approximately 50 mL at 12 weeks, 400 mL at 20 weeks, and reaches almost 1 L at 36-38 weeks. Thereafter, the volume reduces to 600-800 mL at term. It further decreases to approximately 200 mL at 43 weeks [1]. The fluid acts as a shock absorber and protects the fetus from extraneous injuries. Additionally, it allows growth and free movement of the fetus. Amniotic fluid guards against umbilical cord compression and its aseptic and bactericidal action protect the fetus and prevent infection in the uterine cavity [1]. Amniotic fluid is an indicator of placental function and is used for assessing fetal well-being. Amniotic fluid index (AFI) is calculated by adding the depth in centimeters of the largest vertical pocket in each of the four equal uterine quadrants. AFI of ≤5 cm is defined as oligohydramnios. Borderline oligohydramnios (BO) is defined as AFI of 5.1-8 cm [2]. Normal AFI ranges between 8.1 and 25 cm.

According to some studies, borderline AFI increases the risk for cesarean delivery because of fetal distress and increases the incidence of low Apgar score, low birth weight (LBW), and increased need for neonatal intensive care unit (NICU) admission [2-4]. However, Luo et al. did not find any such correlation in terms of fetal distress or neonatal mortality. Even though the incidence of cesarean delivery in borderline cervical ripening was not assessed for every patient, it was determined according to the cervical condition of the patient; hence, it was not assessed in the study. AFI was reported to be higher [5]. In their study, Choi et al. failed to find any correlation between borderline AFI and fetal outcomes [6]. Despite several studies, the prediction of adverse pregnancy outcomes in the presence of borderline AFI is not definite, and, therefore, prenatal surveillance is not recommended in borderline AFI [2]. Given contradictory evidence regarding perinatal and maternal outcomes in the presence of borderline AFI, we decided to conduct this study. We aimed to investigate the perinatal and maternal outcomes in borderline AFI versus normal AFI.

## Materials and methods

This observational prospective study was conducted in a tertiary care center, Pradyumna Bal Memorial Hospital, Kalinga Institute of Medical Sciences, Bhubaneswar, Odisha, a state in the eastern part of India. The study was conducted from September 2019 to February 2021. Institutional Ethics Committee approval was obtained before commencing the study. Patients were recruited in the antenatal clinic after applying inclusion and exclusion criteria.

Inclusion criteria included women with a singleton pregnancy of gestational age between 28 and 40 weeks who were willing to undergo a vaginal delivery and had no contraindication to it. Exclusion criteria included pregnant women with multiple gestations, PPROM, polyhydramnios, and severe oligohydramnios. All eligible patients willing to participate in the study were enrolled. After obtaining informed consent and thorough history, clinical examination, and necessary investigations, an ultrasound examination was performed to assess fetal well-being and determine AFI. Pehlan’s technique was followed while performing ultrasonography (USG) to determine AFI. Obstetric USG with Doppler study (umbilical artery) was done initially to decide the management plan.

The uterus was divided into four imaginary quadrants with the linea nigra and a mediolateral line running through the umbilicus acting as the vertical and the horizontal axes, respectively. The deepest pocket devoid of the umbilical cord and fetal parts was measured in the vertical dimension. The measurement of the four pockets was done in centimeters. The sum of all four quadrants was the AFI value. Patients were divided into two groups according to their AFI score. The study group comprised patients with AFI of 5.1-8 cm, and the control group comprised patients with AFI of 8.1-25 cm. A total of 100 consecutive cases included in each group were investigated.

Fetal surveillance was done with weekly USG and alternate-day cardiotocography (CTG). Doppler velocimetry was carried out during ultrasound examination. Continous intrapartum fetal tracing was done during labor. We excluded all other obstetrics comorbidities and risk factors. Because all cases in the study were not booked, we do not have pre-pregnancy body mass index data for all patients.

Timing and mode of delivery were based on the obstetric indication. Induction of labor was done with syntocinon. For patients in labor, strict intrapartum surveillance was performed. Maternal outcomes included the mode of delivery and term or preterm delivery. Perinatal outcomes included Apgar score, respiratory distress syndrome (RDS), meconium staining of liquor, birth weight, intrauterine growth restriction (IUGR), meconium aspiration, and NICU admission.

SPSS version 23 (IBM Corp., Armonk, NY, USA) was used for statistical analysis. Graphical representation was done in MS Excel 2010. Quantitative data are presented as mean and qualitative data are presented as frequency and percentage. Association between variables was determined using the chi-square test, and p-values of <0.005 were considered to be statistically significant.

## Results

A total of 200 women were enrolled in the study, of which 100 had borderline AFI and 100 had normal AFI. In our study, the mean maternal age was 28.5 years, and there was no statistically significant difference in maternal age in both groups. Borderline AFI was higher in primigravida compared to multigravida women (63% vs. 37%; p-value = 0.002), as shown in Table 1. Admission CTG was reassuring in 71% of cases in the borderline AFI group, whereas it was reassuring in 85% of cases in the normal AFI group.

**Table 1:** Comparison of baseline characteristics. AFI: amniotic fluid index; CTG: cardiotocography

		Case (borderline AFI)	Control (normal AFI)	P-value
Maternal age	<30 years	30	27	0.638
	>30 years	70	73	
Parity	Primigravida	63	41	0.002
	Multigravida	37	59	
Mean gestational age		36 weeks	38 weeks	<0.001
Admission CTG	Reassuring	71	85	-
	Nonreassuring	29	15	-

Occurrence of preterm labour (51% vs. 9%; p < 0.001; odds ratio [OR] = 7.13), meconium-stained liquor (27% vs. 11%; p = 0.006; OR = 2.73), and lower segment cesarean section (LSCS) (59% vs. 35%; p < 0.001), as maternal outcomes, were significantly higher in borderline AFI group, as shown in Table 2.

**Table 2:** Comparison of maternal outcomes. AFI: amniotic fluid index; LSCS: lower segment cesarean section

		Case (borderline AFI)	Control (normal AFI)	P-value
Time of delivery	Preterm	51	9	<0.001
	Term	49	91	
Mode of delivery	LSCS	59	35	<0.001
	Vaginal delivery	41	65	

The most common cause of LSCS in the borderline AFI group was fetal distress, as shown by continuous fetal heart rate monitoring and admission CTG. The most common cause of LSCS in the normal AFI group was nonprogressive labor. Other causes of LSCS are listed in Table 3.

**Table 3:** Indication for cesarean section. MSL: meconium-stained liquor; IUGR: intrauterine growth restriction; CPD: cephalopelvic disproportion

Indication	Cases	Control
Fetal distress	20	6
MSL	10	6
IUGR	15	2
Breech	6	3
Nonprogressive labor	4	8
Failed induction	2	5
CPD	2	5

Neonatal outcomes including Apgar score of <7 at five minutes (35% vs. 14%; p = 0.001; OR = 1.2), RDS (37% vs. 16%; p = 0.001), NICU admission (59% vs. 22%; p < 0.001; OR = 1.93), and IUGR (47% vs. 17%; p < 0.001; OR = 3.37) were also significantly higher in the borderline AFI group. However, meconium aspiration (4% vs. 8%; p = 0.234) was not significantly higher in the borderline AFI group compared to the normal AFI group, as shown in Table 4.

**Table 4:** Comparison of perinatal outcomes. RDS: respiratory distress syndrome; NICU: neonatal intensive care unit; IUGR: intrauterine growth restriction

Neonatal outcomes	Cases (borderline AFI)	Controls (normal AFI)	P-value
Apgar score (<7 at five minutes)	35	14	0.001
RDS	37	16	0.001
NICU admission	59	22	<0.001
IUGR	47	15	<0.001
Baby weight	46	17	<0.001
Meconium-stained liquor	27	11	0.006
Meconium aspiration	4	8	0.234

## Discussion

In the last several years, many studies have been conducted to determine the association between borderline AFI and perinatal outcomes, In most of these studies, rates of maternal and fetal complications were higher in pregnancies with borderline AFI compared to normal AFI [2]. The importance of amniotic fluid volume as an indicator of fetal status with the normal functioning placenta and BO as an indicator of chronic hypoxia is a recent development [7]. Hence, in this study, we compared the maternal and fetal outcomes among women with borderline AFI and normal AFI. We found more adverse perinatal outcomes in women with borderline AFI [8].

In this study, we did not find any statistical difference in the maternal age of the two groups, which is similar to a previous study [8]. The mean maternal age in the previous study was 23.9 years, but in our study, the mean maternal age was 28.5 years. We found that BO was more common in primigravida, which was similar to a previous study; however, in another study, BO incidence was higher in primigravida women [8-10].

In this study, maternal outcomes such as preterm delivery, meconium-stained liquor, and higher rates of LSCS were seen in the borderline AFI group, similar to a previous study [11]. Similarly, poor perinatal outcomes such as Apgar score of <7, RDS, higher NICU admissions, and IUGR were also more in the borderline AFI group, which is consistent with another study reporting a higher incidence of small for gestational age, cesarean section for fetal distress, and NICU admissions in the borderline AFI group [12]. However, another study reported no significant difference in meconium staining, cesarean section rate, LBW, low Apgar score, and the need for NICU admission. There were no neonatal deaths in our study [13]. This may be because of the better NICU facilities available in our institute. We also did not find any congenital malformation in babies in the borderline AFI group, whereas a previous study showed a higher incidence of fetal malformations in the borderline AFI group [14].

Overall, 59% of cases in the borderline AFI group underwent LSCS in our study which was comparable to that reported by Chaudhari et al. [10]. The most common cause of LSCS in the borderline AFI group was fetal distress, as shown by nonreassuring CTG. Moreover, delivery was also significantly higher in the borderline AFI group in our study, which was comparable to another study [14,15]. Pregnant women below the gestational age of <28 weeks were not included as it is associated with poor perinatal outcomes, which confounded the findings of our study of antenatal women with borderline AFI. Additional monetary concern regarding the affordability of NICU is another factor.

A previous study reported an increased rate of cesarean sections and meconium-stained liquor in patients with BO, as well as increased occurrence of LBW (<2.5 kg) and development of fetal distress, which was similar to our study [15].

We found 35% of neonates had Apgar scores of <7 at one minute and 46% had birth weights of <2.5 kg in the borderline AFI group, which are comparable to the study of Manning et al. [17]. The incidence of LBW and IUGR was high possibly because of chronic placental insufficiency. AFI is an indicator of normal placental function and placental insufficiency may lead to decreased fetal growth. Similar findings were also reported by Chaudhari et al. [10]. NICU admissions (59%) were more in the borderline AFI group in our study, which is similar to the findings of Jhonson et al. [18]. In their study, Jamal et al. [4] reported that there was no significant difference in NICU admission and meconium staining among the two groups; however, in our study, we found a significant difference.

The results of our study are not similar to another study that reported no significant difference in fetal distress and Apgar scores in the borderline AFI group compared with the normal AFI group [19]. Another study found a correlation between borderline AFI group and neonatal complications such as low Apgar score, IUGR, LBW, and the need for NICU admission, which was similar to our study [14].

Study limitations

First, because our sample size was small, our results cannot be applied to the whole population. Second, better NICU facilities would have reduced neonatal deaths and resulted in better perinatal outcomes. Finally, fetal distress was diagnosed based on CTG, which can lead to subjective bias, and distress was not confirmed with fetal scalp blood sampling.

## Conclusions

Compared to pregnancies with normal liquor volume, patients with borderline AFI have a higher risk of adverse perinatal outcomes in the form of fetal growth restriction, preterm birth, and major fetal malformations. This is especially true for patients presenting with borderline AFI before 24 weeks of gestation. Hence, there is a need for intensive fetal surveillance during the antepartum period. During labor, proper vigilance and intrapartum monitoring become the key for better fetomaternal outcomes. Every case needs careful antenatal assessment and individualization of the decision regarding the timing and mode of delivery. Future well-controlled trials with larger sample sizes should focus on the evaluation of the management strategy for patients presenting with borderline AFI.
